# Electronic nicotine devices to aid smoking cessation by alcohol- and drug-dependent clients: protocol for a pilot randomised controlled trial

**DOI:** 10.1186/s13063-018-2786-1

**Published:** 2018-08-02

**Authors:** Ashleigh Guillaumier, Victoria Manning, Olivia Wynne, Coral Gartner, Ron Borland, Amanda L. Baker, Catherine J. Segan, Eliza Skelton, Lyndell Moore, Ramez Bathish, Dan I. Lubman, Billie Bonevski

**Affiliations:** 1 School of Medicine & Public Health, University of Newcastle, Callaghan, Australia; 2 Eastern Health Clinical School, Monash University, Box Hill, VIC Australia; 3 Turning Point, Eastern Health, Fitzroy, Australia; 4 Faculty of Medicine, School of Public Health, The University of Queensland, St Lucia, QLD Australia; 5 The Cancer Council Victoria, Melbourne, Victoria Australia; 6 Centre for Health Policy, School of Population and Global Health, University of Melbourne, Melbourne, Victoria Australia

**Keywords:** Electronic cigarettes, Alcohol and other drugs, Smoking cessation, Nicotine replacement therapy

## Abstract

**Background:**

Up to 95% of people entering treatment for use of alcohol or other drugs (AOD) smoke tobacco. Smokers receiving treatment for AOD use are interested in quitting and make quit attempts, but relapse is more common and rapid compared with the general population of smokers. New ways to address smoking in this population are needed. Electronic nicotine devices (ENDs) or electronic cigarettes hold significant potential as both cessation aids and harm reduction support. This study focuses on the potential of ENDs to facilitate smoking cessation and to sustain it in the medium term among people in treatment for AOD use. The aim of this trial is to explore the effectiveness, feasibility and acceptability of ENDs for smoking cessation compared with combination nicotine replacement therapy (NRT) for clients after discharge from a smoke-free AOD residential withdrawal service.

**Methods/design:**

The study is a pragmatic randomised controlled trial. In total, 100 participants will be recruited following admission to a smoke-free residential withdrawal service in Melbourne, Australia. Participants will complete a baseline survey and be randomised to either the END group (*n* = 50) or the NRT group (*n* = 50) prior to discharge. Both groups will receive telephone counselling support from quitline. Follow-up measures will be assessed at 6 and 12 weeks following discharge. The primary outcome is continuous abstinence from smoking at 12 weeks post discharge. Secondary outcomes include: 7-day point prevalence from smoking, point prevalence abstinence from all nicotine (including NRT and ENDs), cravings and withdrawal, time to relapse, and treatment adherence (use of NRT, ENDs and quitline).

**Discussion:**

This is the first randomised controlled trial to assess the effectiveness and acceptability of ENDs within a population dependent on AOD, a priority group with very high levels of smoking. The research will test a model of how to incorporate novel smoking cessation support into a period of high treatment receptiveness.

**Trial registration:**

Australian New Zealand Clinical Trial Registry, ACTRN12617000849392. Registered on 8 June 2017.

**Electronic supplementary material:**

The online version of this article (10.1186/s13063-018-2786-1) contains supplementary material, which is available to authorized users.

## Background

In Australia, up to 95% of people entering treatment for use of alcohol or other drugs (AOD) smoke tobacco, which is five times more than for the general adult population [1]. A recent large international systematic review (*n* = 37,364) showed that smoking rates among people in treatment for AOD use (84%) are more than double those for people with similar demographic characteristics in the general population (31%) [2]. As a result, people in treatment for AOD use experience a greater tobacco-related disease burden.

People with a mental illness and substance dependence die 25 years earlier than those without such disorders, and the main causes of death are tobacco-related conditions, including cancer, cerebrovascular diseases and chronic respiratory diseases [3]. In fact, smokers with comorbid substance dependence are more likely to die from tobacco-related causes than from other substance-related causes [4, 5], and quitting smoking is associated with longer-term maintenance of recovery from other addictions [6]. In addition to the health effects of tobacco, smoking causes significant financial stress and social isolation among this already disadvantaged group [7]. Smokers receiving treatment for AOD use are interested in quitting and make quit attempts [8]. The number of quit attempts is high, since sustaining cessation is challenging for people with AOD dependence.

While 85% of quit attempts using standard medication and behavioural support fail in the long term (e.g. 12 months) among the general population of smokers [9], this rate is even higher among smokers with AOD use [10]. High relapse rates in this population may be due to factors related to addiction (e.g. smoking-related cues and triggers), lack of cessation support and high levels of smoking in their social network [1, 11]. Three large trials providing AOD clients with traditional behavioural support and nicotine replacement therapy (NRT) achieved short-term (8–12 weeks) abstinence rates between 9% and 33% while in treatment, but most relapsed to smoking following discharge [12–14]. The heavy nicotine dependence among smokers with AOD use is an important contributor to this high relapse rate [8]. NRT is standard practice as a smoking cessation aid for the general population [15] and is recommended in guidelines Australia [16] and other countries [17, 18] for AOD inpatient services. NRT can reduce withdrawal symptoms and cravings and aid cessation. However, our research with AOD users indicates that they are hesitant to use traditional forms of NRT [19]. New ways of thinking about how to address smoking among this population are needed.

### Electronic nicotine devices

Electronic nicotine devices (ENDs) hold significant potential as both cessation aids and harm reduction support. ENDs are a broad range of battery-powered devices that deliver an aerosol of propylene glycol and/or glycerine, nicotine and flavours [20]. Unlike combustible tobacco cigarettes, ENDs deliver nicotine in an inhalable form without burning tobacco. This is an important development, because the vast majority of the health harms of smoking are due to the toxins produced when tobacco is burnt, rather than the nicotine. Like traditional NRT, the provision of nicotine in END vapour reduces cravings and withdrawal symptoms. Furthermore, ENDs also address the behavioural aspects of smoking cigarettes, such as the hand-to-mouth action, and the inhaling and exhaling of “smoke” (vapour) [21]. With practice, users of advanced ENDs can obtain similar blood nicotine levels to cigarette smokers [22]. END aerosol may still contain some toxins, especially if the liquid is overheated, but the number of toxins is much lower than in cigarette smoke and those that are present are at much lower concentrations [23]. A recent extensive review of the scientific evidence on ENDs concluded that they are likely to be around 95% less harmful than cigarettes [23]. Many smokers report using ENDs to help them quit using tobacco cigarettes [24, 25] and smokers with strong intentions to quit are significantly more likely to have used ENDs than smokers with no intention to quit [25]. Further, those who use e-cigarettes daily were significantly more likely to have stopped using cigarettes compared to those who have never tried e-cigarettes [26].

A recent Cochrane review found that ENDs are effective, but not necessarily more effective than other forms of NRT [27]. The authors concluded that this was due to a lack of evidence and recommended more studies be conducted. Trials with smokers in the general population have demonstrated the safety and efficacy of ENDs compared to other forms of NRT. In New Zealand, one study found that verified abstinence rates at 6 months were 7.3% for early-generation low-nicotine-delivery ENDs, 5.8% for NRT patches and 4.1% for placebo ENDs [28]. Cigarette consumption was reduced by at least half in 57% of the participants allocated to nicotine ENDs vs 41% in the patches group and 45% in the placebo group. Median time to relapse was twice that in participants allocated to nicotine ENDs than patches or the placebo. No significant differences in adverse events were observed. A secondary analysis of participants with mental illness found no differences between END and NRT for rates of relapse, but the e-cigarette group had higher levels of smoking reduction, treatment adherence and acceptability [29]. Further, a longitudinal study in the USA found that at the 2-year follow-up, long-term ENDs users had a higher cessation rate (42.4%) than short-term users (14.2%) or non-users (15.6%). Also, for those participants reporting quit attempts, significantly more reported using ENDs as a cessation aid (24.8%) than reported using an FDA-approved NRT (17.8%) [30]. A similar study with people on methadone maintenance therapy found no difference in the effectiveness of e-cigarettes and NRT [29]. Another in people living with mental illness found self-reported combustible tobacco use and breath CO significantly declined among those using an e-cigarette [30]. However, most studies so far have been with small participant numbers or have focused on short-term timeframes. More evidence gathered from well-controlled studies with larger sample sizes to inform policy and practice regarding END use is needed, particularly of the likelihood that alternative forms of nicotine will be taken up as cessation aids or harm reduction tools, and with populations with high smoking prevalence and low quit rates.

## Methods/design

### Aim

The aim of this pilot pragmatic randomised controlled trial is to explore the feasibility, acceptability and effectiveness of providing an END vaping kit with a 12-week supply of liquid nicotine and telephone quitline support compared with a 12-week supply of combination NRT and quitline support to clients upon discharge from a smoke-free AOD residential withdrawal service.

### Design

**Fig. 1:**
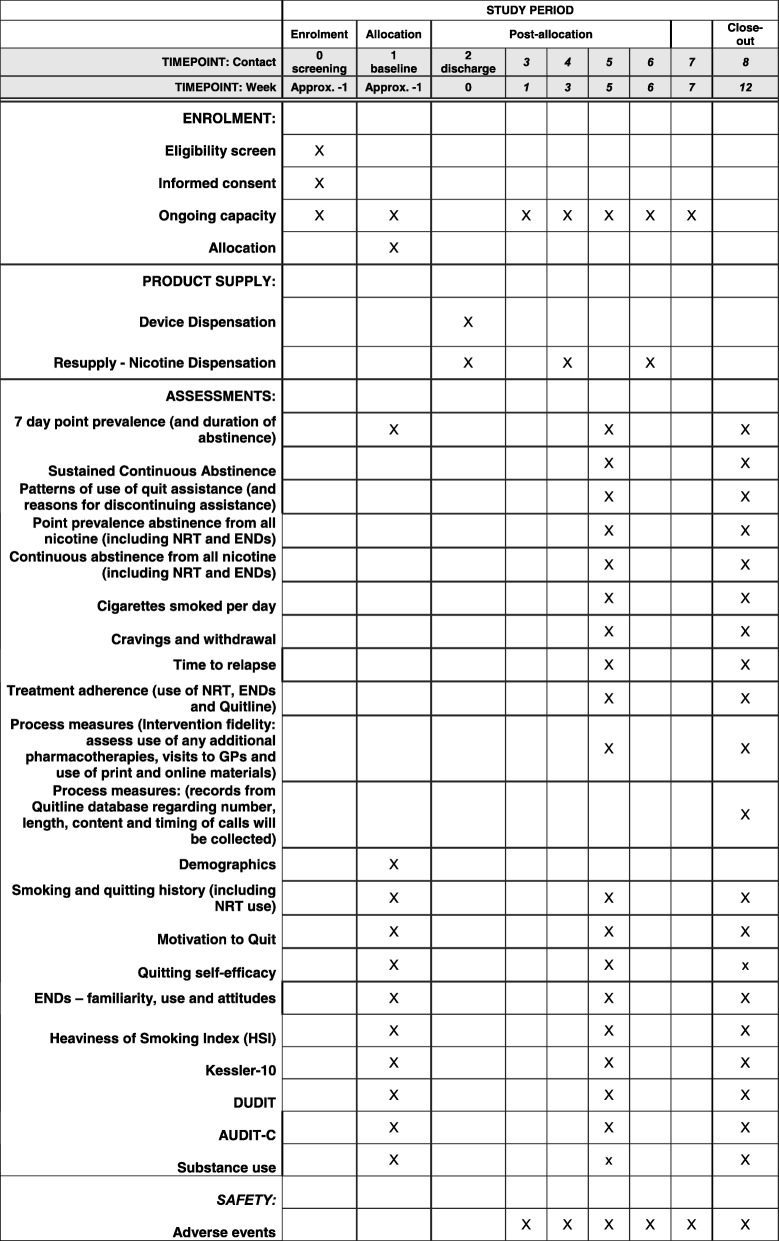
Schedule of enrolment, product supply and assessments. DUDIT Drug Use Disorders Identification Test, END electronic nicotine device, GP general practitioner, NRT nicotine replacement therapy

The study uses a pragmatic design. It is a phase II, open-label, active control, single-centre, exploratory trial of the effectiveness, feasibility and acceptability of ENDs and liquid nicotine plus quitline counselling among AOD clients discharged from care compared to current best practice (combination NRT and quitline counselling) for smoking cessation and relapse prevention. Follow-up measures will be assessed at 6 and 12 weeks after discharge from the service. Due to the nature of the intervention, neither participants nor staff can be blinded to allocation. However, the data safety monitoring committee and the statistician responsible for the data analysis will be blinded. This trial is registered with the Australian New Zealand Clinical Trials Registry (ACTRN12617001205325p). This study has received ethics approval from the Eastern Health human research ethics committee (E16–2016) and the University of Newcastle human research ethics committee (H-2017-0249). The trial follows the recommendations for interventional trials guidelines (SPIRIT; see Additional file 1). The study schedule of enrolment, product supply and assessments is presented in Fig. 1.

### Setting

Participants will be recruited from a 12-bed residential AOD withdrawal service in Melbourne, Australia. The length of stay is 7–10 days, with an average of 8 days. It is staffed by health professionals who provide medical supervision, support, educational and recovery-focused groups, treatment and discharge planning. The site is smoke-free and as part of current usual care, all clients receive NRT while in the programme to assist with the management of their nicotine withdrawal symptoms, but not following discharge.

### Participants

#### Inclusion criteria

Individuals must meet all the following criteria to be enrolled in this study:

- aged 18 years or over
- tobacco smoker on entering the residential service
- have the capacity to consent and able to understand the participant materials and follow the study instructions and procedures (e.g. sufficient English language ability)

#### Exclusion criteria

Individuals who meet any the following criteria will not be enrolled in this study:

- have used an END containing nicotine in the past month
- currently pregnant or breast-feeding (measured by self-report)
- currently enrolled in another study
- scheduled to be transferred to a long-term rehabilitation unit following discharge from the residential withdrawal unit

### Procedure

**Fig. 2:**
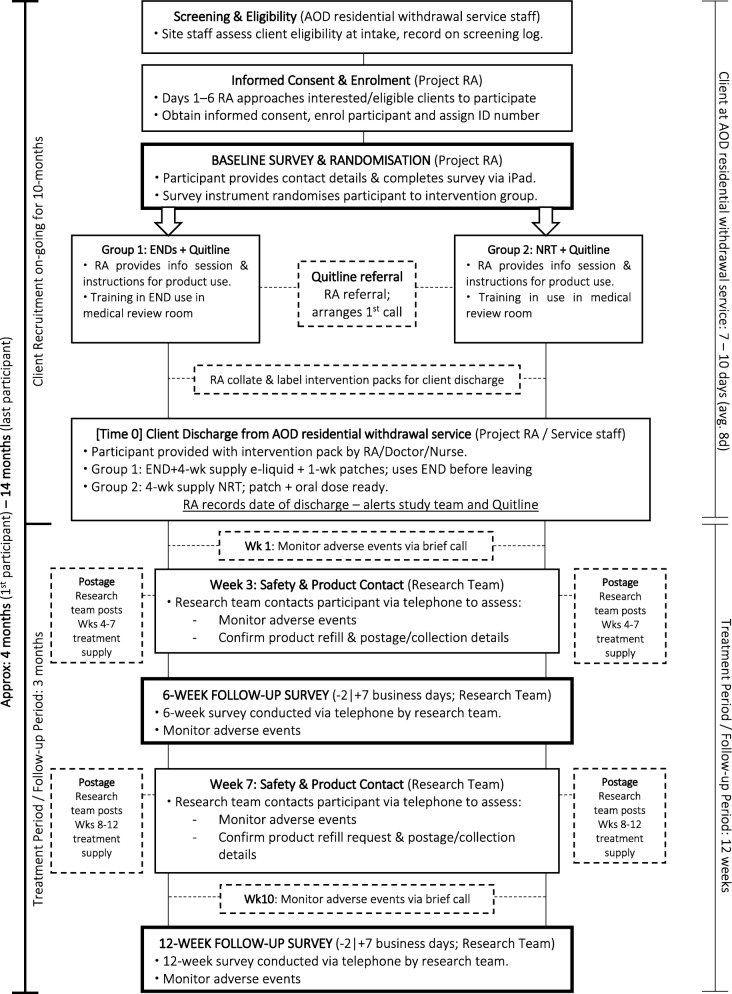
Flow chart of participant recruitment and follow-up. AOD alcohol or other drugs, END electronic nicotine device, NRT nicotine replacement therapy, RA research assistant, Wk week

During the intake assessment, residential withdrawal unit staff will screen clients for eligibility and notify eligible clients that a research trial is being conducted and ask if they would be interested in receiving more information about the trial from a research assistant (RA) on site. Unit staff will notify the RA which clients are interested in being approached about participating in the project. The RA will approach interested clients 1–6 days post intake, explain the project, provide written participant information sheets and seek written consent. Figure 2 outlines participant recruitment and follow-up.

All potential participants will receive a copy of the participant information and consent form and the project RA will give a full verbal explanation of the trial (aims, procedures, risks and benefits) in lay terms. Potential participants will have the opportunity to discuss the trial and ask questions. Participants will be advised they can withdraw from the study at any time without prejudice and that their decision to participate in the trial or not will in no way affect the care they receive at the residential withdrawal unit. Following this process, the participant will be asked to provide written consent to participate in the study and complete the baseline survey. Consent will also be collected for the research team to use participant contact details at the end of the trial to possibly invite the participant to take part in a one-off telephone interview about their study experience.

After formal enrolment into the trial, participants will be assigned a unique study ID number, which will be in a re-identifiable format. The ID number will be used by the secure online survey platform, into which participants will enter their identifying information (follow-up contact details) and then proceed on to the baseline questionnaire items. The participant-identifying details will be stored separate to other data, while the ID number will be attached to both. Collection of the baseline data will be completed by the participant online via an iPad. At the end of the baseline survey, participants will be randomised 1:1 to an intervention via a computer-sequenced 4–6 block randomisation embedded in the iPad.

Following randomisation, the project RA will notify participants of the intervention group they have been assigned to and take them through the contents of the relevant intervention pack. Both groups will receive:

- Proactive referral to quitline counselling (call-back service), which provides calls at pre-discharge and on days 1, 3, 7, 14 and 28 post-discharge, with an emphasis on relapse prevention. At least one call will be scheduled during their residential withdrawal stay. The total number and timing of calls will be tailored to client need and smoking status (i.e. with more frequent calls around relapse crises and quit attempts). Participants will be sent a text message prior to being called, since AOD clients are unlikely to pick up calls from a private number. Counsellors will be trained on the use of ENDs, will monitor and encourage correct use of NRT and will work with clients to address barriers to their use.
- A trial business card that contains the study’s toll-free number

The END group will receive:

- An information pack that includes printed information on the benefits of quitting (including that quitting tobacco may facilitate AOD reduction) and information about nicotine maintenance, the benefits of vaping instead of smoking and the risks of vaping compared to complete abstinence. The lack of data on th health risks of long-term vaping will be highlighted. Instructions on how to use ENDs and safe storage and handling will be included.
- A 1-week supply of nicotine patches for use while getting used to the END.
- An END starter kit. The device (Innokin Endura T22 starter kit) and refill liquid (Nicophar) were selected based on quality assurance and compliance with relevant standards.
- A 4-week supply of liquid nicotine, with further supplies of liquid nicotine mailed to them twice at 4-week intervals.

The NRT group will receive:

- An information pack like that provided to the END group, but rather than information on ENDs, it will include instructions on how to use NRT correctly and for how long, plus information on potential side effects (and when to notify a health-care provider), safe storage and handling.
- Provision of 12 weeks of NRT on the same schedule as for ENDs. They will initially receive a 4-week supply of patches plus a nicotine spray and inhaler, which will be followed by refills including patches plus inhaler, gum and lozenges.

Participants in both groups will be contacted after discharge at 3 weeks and again at 7 weeks to confirm if they would like to receive a second and third supply of the relevant product (for weeks 5–8 and 9–12, respectively).

### Dose modification

For the END group, the dosing schedule of e-liquid provided to participants will be dependent on their nicotine dependence score as measured by the Heaviness of Smoking Index [31, 32]. Participants scoring in the high-nicotine-dependence category will be assigned an initial 4-week e-liquid supply (total 8 × 10 ml bottles) consisting of: 2 × 10 ml bottles of 18 mg e-liquid and 6 × 10 ml bottles of 12 mg e-liquid. This allotment allows for a 1-week supply of 18 mg e-liquid while participants in this group familiarise themselves with the use of the device. The second and third batches of e-liquid will consist of 8 × 10 ml bottles of 12 mg e-liquid only. Participants scoring in the moderate- and low-dependence categories will receive three 4-week supplies of 8 × 10 ml bottles of 12 mg e-liquid.

### Outcomes

#### Primary outcome

The primary outcome is the continuous abstinence from smoking (defined using a modified version of the Russell standard [33]), that is, no more than five (tobacco) cigarettes after their discharge from the residential AOD withdrawal service. Sustained abstinence from discharge will be checked at each follow-up time point, i.e. by self-report at weeks 6 and 12. Sustained abstinence will be assessed from the date of the previous interview: “Since [date] did you smoke at all, even part of a cigarette?” and among those who did: “In the past 6 weeks (that is, since [date]), have you smoked a tobacco cigarette, even a puff?” with the response options: (1) no, not a puff, (2) 1–5 cigarettes or (3) more than five cigarettes.

#### Secondary outcomes

Secondary outcome measures assessed at 6 and 12 weeks, include:

i. 7-day point prevalence smoking abstinence, based on: “Have you smoked at least part of a cigarette in the last 7 days?”
ii. Point prevalence abstinence from all nicotine (including NRT and ENDs), defined as having not used any products containing nicotine in the previous 7 days at assessment.
iii. Continuous abstinence post discharge from all nicotine (including NRT and ENDs).
iv. Reported number of cigarettes smoked per day among those who relapse (a relapse is defined as 7 days of continuous smoking for previously daily smokers).
v. Cravings, assessed by one item based on Taggar et al. [34]: “Currently, how often do you get strong cravings to smoke tobacco?” with the response options: (1) hourly or more often, (2) several times per day, (3) at least once a day or (4) less than daily.
vi. Withdrawal, as assessed by the Minnesota Nicotine Withdrawal Scale [35, 36], an eight-item scale rating withdrawal symptoms on an ordinal scale ranging from 0 (not present) to 4 (severe).
vii. Time to relapse, which will be determined for those who do relapse by asking when they first smoked after a quit attempt.
viii. Feasibility, which will be assessed by collecting the recruitment rate, consent rate and attrition rate.
ix. Acceptability, by asking about the extent of use of the nicotine products provided. This will be determined by a five-point Likert scale administered in the 6- and 12-week surveys. The following question will be asked: “Thinking about your use of and experience with the [product], please indicate your agreement with the following statements: it was effective at reducing my cravings; it was easy to use; it was enjoyable to use.” The response options are: (1) strongly disagree, (2) disagree, (3) undecided, (4) agree or (5) strongly agree. Those in the NRT group will be asked about each type of product individually.
x. Number of subsequent quit attempts among those who relapsed.
xi. Any adverse events reported during the study.
xii. Treatment adherence (use of NRT, ENDs and quitline). Adherence to treatment will be measured by questions about product use in the 6- and 12-week surveys. Participants will be asked if they have used the products and if they are currently using the products. If they are not using the products, then they will be asked why, otherwise they all be asked about the frequency of use and if they use a combination of products.
xiii. Process measures. The 6- and 12-week surveys will assess the use of any additional pharmacotherapies, general practice visits and the use of printed and online materials to aid in quitting. Records from the quitline database regarding number, length, content and timing of calls will also be collected. In addition, cost data will be recorded.

### Covariate measures

A number of covariate measures will be used for the statistical analysis, including:

i. Demographic variables such as gender.
ii. History of tobacco smoking and quitting.
iii. Motivation to quit, assessed by a 10-point Likert scale where 1 is very low and 10 is very high [37].
iv. Quitting self-efficacy, which is assessed using the following item: “If you decided to give up smoking completely in the next 6 months, how sure are you that you would succeed?” with response options: (1) not at all sure, (2) slightly sure, (3) moderately sure, (4) very sure and (5) extremely sure [38].
v. Heaviness of smoking index: Nicotine dependence is assessed using the two-item Index [31, 32]. It uses a six-point scale calculated from the number of cigarettes smoked per day (1–10, 11–20, 21–30 or 31+) and the time to first cigarette after waking (≤5, 6–30, 31–60 or 61+ minutes). Nicotine dependence is then categorised into a three-category variable: low (0–1), medium (2–4) or high (5–6).
vi. Alcohol Use Disorders Identification Test: Brief (AUDIT-C) [39, 40], which is a three-item screening tool used to identify hazardous alcohol use or active alcohol use disorders. It is scored on a scale of 0–12 with a cut-off of 3 (women) or 4 (men). For men, it has been shown to have a sensitivity of .90 and specificity of .45. For women, the sensitivity is .80 and specificity is .87 [39, 41].
vii. Drug Use Disorders Identification Test (DUDIT) [42], an 11-item instrument designed to parallel AUDIT-C with a scale of 0–44. It has been shown to have a sensitivity of .90 and a specificity of .88, with a Cronbach’s alpha coefficient of .80 [42].
viii. Kessler Psychological Distress Scale (Kessler-10) [43], a 10-item scale of non-specific psychological distress with low scores (10–15) indicating little or no psychological distress, moderate scores (16–21), high scores (22–29) and very high scores (30–50) indicating increasing levels of distress.

### Statistical methods

#### Sample size estimation

We expect to recruit 100 clients over a 10-month period. Approximately 80% of clients to the service are smokers. We will be assuming 50% eligibility and consent into the trial, and 40% attrition at 12 weeks based on our current AOD research. A total of 60 participants at the 12-week follow-up will provide 30 smokers per experimental group. This trial has been designed to provide essential preliminary information for the development of an adequately powered larger superiority trial, including smoking prevalence, response and attrition rates.

#### Statistical analysis plan

Data will be collected on recruitment and retention rates, which will aid the design of future larger trials. Primary cessation outcome analyses will be carried out on an intention-to-treat basis. We will use chi-squared tests to compute relative risks, 95% confidence intervals and two-sided *p* values for all binary variables, followed by adjusted multiple logistic regression analyses. Continuous outcomes (with 95% confidence intervals) will be analysed using generalised linear mixed methods regression for the main aims, with adjustments for covariates where necessary.

## Discussion

This randomised controlled trial will be the first to assess the effectiveness, feasibility and acceptability of ENDs within an AOD-dependent population in Australia. This study is targeting a high-priority group with a very high prevalence of smoking. In the short term, the project will provide essential information for the design of a larger superiority trial, which will be powered to assess the safety and efficacy of ENDs and/or NRT use for sustained cessation and harm reduction outcomes in AOD clients.

AOD-dependent people smoke at higher rates than the general population [1, 2] and suffer from a greater smoking-related disease burden [4, 5, 7]. This vulnerable population should be a target for harm reduction research as a public health priority. Further, clear-air laws mean that most residential treatment services are smoke-free. Patients in the services are required to abstain from combustible tobacco, and most services supply NRT in the form of patches for the duration of the stay. While many patients express a desire to quit, most report returning to smoking immediately after discharge from inpatient services [8]. Thus, the inpatient period is a unique window of opportunity for patients to experience forms of nicotine delivery other than combustible tobacco.

ENDs are a relatively new product with a rapid rise in use in the general population [44]. Research on the acceptability and feasibility of ENDs as an alternative to tobacco is relatively limited. However, the devices are considered to be a less harmful alternative to combustible tobacco [23], and have been found to improve cessation rates [26]. The particular devices used in the study were selected due to their relative affordability and reliability as well as ease of use, and they were not manufactured by a tobacco company. The strength of the e-liquid supplied is similar to a liquid nicotine product currently listed on the Australian Register of Therapeutic Goods, which is approved for over-the-counter sale in general retail outlets, Nicorette QuickMist Mouthspray. It contains 13.6 mg/mL of nicotine and each pack contains 13.2 mL (i.e. the total nicotine per pack is 179.5 mg). The 4-week supply of 8 × 10 ml bottles of liquid, a total supply of 80 ml, equates to approximately 3 ml of liquid per day, which is considered an appropriate amount of nicotine to be prescribed for such devices [45]. Recent studies suggest that devices such as those to be used in the current study have better nicotine delivery than existing NRT products [22, 46]. However, note that the specific END in the study is not currently approved by the Therapeutic Goods Administration for therapeutic use in Australia.

Another facet of the project is the referral to a quitline telephone counselling service. Telephone quitlines are low cost and convenient to access, they can be tailored to individuals, and they have the potential to reach a broad population of smokers. Australia has a national quitline service, with a common telephone number in each state and territory. The service provides evidence-based information and assistance in a single call (reactive) as well as repeated calls from trained counsellors (proactive). A Cochrane review found that quitlines are effective for the general population and that call-back counselling (proactive) enhances their usefulness (nine studies, >24,000 participants, relative risk 1.37 and 95% confidence interval 1.26–1.50) [47]. However, the utilisation of quitline services is low. For example, in Australia, it is estimated that only 3.2–3.6% of smokers access the service [48, 49]. Furthermore, there is no information about the engagement of the AOD population with quitlines. The proposed study will provide data on the engagement and acceptability of the quitline within this hard to reach group of smokers.

Possible challenges for the study include recruitment and retention rates. The AOD population is considered hard to reach and difficult to retain [50]. The reasons for the difficulty in retention are systemic, with disadvantages due to sociological and economic factors, as well as high comorbidity being an issue for the population. However, if the overall aim of health research is reducing health burden and increasing equity, it is those systemic issues that should drive research with this vulnerable population rather than deter it. Recruitment rates in this population may be low. In the proposed study, we aim to mitigate the issue by offering incentives in line with the project objectives (free NRT or ENDs), and including detailed information in the recruitment phase (benefits of quitting and requirements of participation). To control for attrition, we plan regular scheduled contact with participants over the course of the study, as well as gathering collateral contact details (up to four secondary contacts), and liaising with residential treatment services to maintain contact with participants who may go into long-term residential rehab during the study period.

The current study is not powered to detect small differences in cessation; however, it will provide valuable information on the feasibility, acceptability and potential effectiveness of ENDs as a smoking cessation and harm reduction aid with AOD populations, and inform the design of a larger, powered study. It will also provide safety and adverse events data. The project also lacks a no-intervention placebo, control group. However, the combination NRT group represents current best practice and this study was designed to test the feasibility of ENDs in this population compared with current best practice and to inform future larger-scale studies. Another limitation is that the study utilises self-reported cessation rather than any biomarker confirmation. Biochemical verification is recommended for studies using the Russell standard as an outcome measure [51]; however, experts have agreed that biochemical validation methods need not always be used to validate self-report smoking cessation measures [52]. While it is possible to include biochemical verification in a trial such as this, the limited funding available made it difficult to do so. Following consultation between all study investigators, it was decided that given the aims of the pilot trial were to test the acceptability and feasibility of ENDs, and the study is not powered to determine the effectiveness of smoking cessation (which would be the primary reason for biochemical verification), that the additional cost and resources (in staff time) required to complete the biochemical verification were not warranted. Note that the study authors have submitted a funding application for an adequately powered effectiveness randomised controlled trial, which will assess biochemically verified smoking cessation as the primary aim.

The trial will provide insights into the potential viability of providing smoking cessation interventions as clients leave a smoke-free residential setting. This is important because most resume smoking, typically as soon as they can access cigarettes [8]. In the longer term, this research will contribute to the evidence base to inform policy development regarding the adoption of smoking cessation and tobacco harm reduction approaches within the AOD treatment setting. Harm reduction is an approach commonly used within AOD treatment settings; however, it has not been applied empirically for tobacco smoking. The research will provide a model for how to incorporate cessation support in AOD settings, particularly residential smoke-free services, which present a unique opportunity for intervention and the adoption of harm reduction approaches.

### Trial status

The current protocol version is 3.0, dated 11 January 2017. Recruitment began on 1 August 2017. Recruitment is expected to be completed by about July 2018.

## Additional file

Additional file 1: SPIRIT checklist. (DOC 121 kb)

## Abbreviations

AOD: Alcohol or other drugs
AUDIT-C: Alcohol Use Disorders Identification Test: Brief
DUDIT: Drug Use Disorders Identification Test
END: Electronic nicotine device
NRT: Nicotine replacement therapy
RA: Research assistant

## References

[1] Baker A, Ivers RG, Bowman J, Butler T, Kay-Lambkin FJ, Wye P, Walsh RA, Pulver LJ, Richmond R, Belcher J (2006). Where there's smoke, there's fire: high prevalence of smoking among some sub-populations and recommendations for intervention. Drug Alcohol Rev.

[2] Guydish J, Passalacqua E, Pagano A, Martinez C, Le T, Chun J, Tajima B, Docto L, Garina D, Delucchi K (2016). An international systematic review of smoking prevalence in addiction treatment. Addiction.

[3] Colton CW, Manderscheid RW (2006). Congruencies in increased mortality rates, years of potential life lost, and causes of death among public mental health clients in eight states. Prev Chronic Dis.

[4] Randall D, Degenhardt L, Vajdic CM, Burns L, Hall WD, Law M, Butler T (2011). Increasing cancer mortality among opioid-dependent persons in Australia: a new public health challenge for a disadvantaged population. Aust N Z J Public Health.

[5] Taylor Hays J, Schroeder DR, Offord KP, Croghan IT, Patten CA, Hurt RD, Jorenby DE, Fiore MC (1999). Response to nicotine dependence treatment in smokers with current and past alcohol problems. Ann Behav Med.

[6] McKelvey K, Thrul J, Ramo D (2017). Impact of quitting smoking and smoking cessation treatment on substance use outcomes: an updated and narrative review. Addict Behav.

[7] Guillaumier A, Twyman L, Paul C, Siahpush M, Palazzi K, Bonevski B. Financial stress and smoking within a large sample of socially disadvantaged Australians. Int J Environ Res Public Health. 2017;14(3):231. 10.3390/ijerph14030231 10.3390/ijerph14030231 PMC5369067 28245612

[8] Kelly PJ, Baker AL, Deane FP, Kay-Lambkin FJ, Bonevski B, Tregarthen J (2012). Prevalence of smoking and other health risk factors in people attending residential substance abuse treatment. Drug Alcohol Rev.

[9] Ferguson J, Bauld L, Chesterman J, Judge K (2005). The English smoking treatment services: one-year outcomes. Addiction.

[10] Richter KP, Arnsten JH (2006). A rationale and model for addressing tobacco dependence in substance abuse treatment. Subst Abuse Treat Prev Policy.

[11] Twyman L, Bonevski B, Paul C, Bryant J. Perceived barriers to smoking cessation in selected socioeconomically disadvantaged groups: a systematic review of the qualitative and quantitative literature. BMJ Open. 2014;4:1–15. 10.1136/bmjopen-2014-006414 10.1136/bmjopen-2014-006414 PMC4275698 25534212

[12] Reid MS, Fallon B, Sonne S, Flammino F, Nunes EV, Jiang H, Kourniotis E, Lima J, Brady R, Burgess C (2008). Smoking cessation treatment in community-based substance abuse rehabilitation programs. J Subst Abus Treat.

[13] Shoptaw S, Rotheram-Fuller E, Yang X, Frosch D, Nahom D, Jarvik ME, Rawson RA, Ling W (2002). Smoking cessation in methadone maintenance. Addiction.

[14] Stein MD, Weinstock MC, Herman DS, Anderson BJ, Anthony JL, Niaura R (2006). A smoking cessation intervention for the methadone-maintained. Addiction.

[15] RACGP (2011). Supporting smoking cessation: a guide for health professionals.

[16] NSW Ministry of Health (2002). The guide for the management of nicotine dependent inpatients.

[17] Fiore M (2008). Treating tobacco use and dependence: 2008 update: clinical practice guideline.

[18] Ratschen E, Britton J, McNeill A (2011). The smoking culture in psychiatry: time for change. Br J Psychiatry.

[19] Wilson AJ, Bonevski B, Dunlop A, Shakeshaft A, Tzelepis F, Walsberger S, Farrell M, Kelly PJ, Guillaumier A (2016). 'The lesser of two evils': a qualitative study of staff and client experiences and beliefs about addressing tobacco in addiction treatment settings. Drug Alcohol Rev.

[20] Gartner C, Hall W (2015). A licence to vape: is it time to trial of a nicotine licensing scheme to allow Australian adults controlled access to electronic cigarettes devices and refill solutions containing nicotine?. Int J Drug Policy.

[21] Rooke C, Cunningham-Burley S, Amos A (2016). Smokers’ and ex-smokers’ understanding of electronic cigarettes: a qualitative study. Tob Control.

[22] St Helen G, Havel C, Dempsey DA, Jacob P, Benowitz NL (2016). Nicotine delivery, retention and pharmacokinetics from various electronic cigarettes. Addiction.

[23] McNeill A, Brose LS, Calder R, Hitchman SC, Hajek P, McRobbie H. E-cigarettes: an evidence update. A report commissioned by Public Health England. London: Public Health England; 2015.

[24] Phung A, Luo L, Breik N, Alessi-Severini S (2017). Use of smoking cessation products: a survey of patients in community pharmacies. Can Pharm J/Revue des Pharmaciens du Canada.

[25] Adkison SE, O'Connor RJ, Bansal-Travers M, Hyland A, Borland R, Yong HH, Cummings KM, McNeill A, Thrasher JF, Hammond D, Fong GT (2013). Electronic nicotine delivery systems: international tobacco control four-country survey. Am J Prev Med.

[26] Giovenco DP, Delnevo CD (2018). Prevalence of population smoking cessation by electronic cigarette use status in a national sample of recent smokers. Addict Behav.

[27] Hartmann-Boyce J, McRobbie H, Bullen C, Begh R, Stead LF, Hajek P. Electronic cigarettes for smoking cessation. Cochrane Database Syst Rev. 2016;9:CD010216. 10.1002/14651858.CD010216.pub3 PMC6457845 27622384

[28] Bullen C, Howe C, Laugesen M, McRobbie H, Parag V, Williman J, Walker N (2013). Electronic cigarettes for smoking cessation: a randomised controlled trial. Lancet.

[29] O’Brien B, Knight-West O, Walker N, Parag V, Bullen C (2015). E-cigarettes versus NRT for smoking reduction or cessation in people with mental illness: secondary analysis of data from the ASCEND trial. Tob Induc Dis.

[30] Zhuang YL, Cummins SE, Sun JY, Zhu SH (2016). Long-term e-cigarette use and smoking cessation: a longitudinal study with US population. Tob Control.

[31] Heatherton TF, Kozlowski LT, Frecker RC, Rickert W, Robinson J (1989). Measuring the heaviness of smoking: using self-reported time to the first cigarette of the day and number of cigarettes smoked per day. Br J Addict.

[32] Kozlowski LT, Porter CQ, Orleans CT, Pope MA, Heatherton T (1994). Predicting smoking cessation with self-reported measures of nicotine dependence: FTQ, FTND, and HSI. Drug Alcohol Depend.

[33] West R, Hajek P, Stead L, Stapleton J (2005). Outcome criteria in smoking cessation trials: proposal for a common standard. Addiction.

[34] Taggar JS, Lewis S, Docherty G, Bauld L, McEwen A, Coleman T (2015). Do cravings predict smoking cessation in smokers calling a national quit line: secondary analyses from a randomised trial for the utility of 'urges to smoke' measures. Subst Abuse Treat Prev Policy.

[35] Toll BA, O’Malley SS, McKee SA, Salovey P, Krishnan-Sarin S (2007). Confirmatory factor analysis of the Minnesota nicotine withdrawal scale. Psychol Addict Behav.

[36] Patten CA, Martin JE (1996). Measuring tobacco withdrawal: a review of self-report questionnaires. J Subst Abus Treat.

[37] Zhou X, Nonnemaker J, Sherrill B, Gilsenan AW, Coste F, West R (2009). Attempts to quit smoking and relapse: factors associated with success or failure from the ATTEMPT cohort study. Addict Behav.

[38] Fidler JA, Shahab L, West O, Jarvis MJ, McEwen A, Stapleton JA, Vangeli E, West R (2011). 'The smoking toolkit study': a national study of smoking and smoking cessation in England. BMC Public Health.

[39] Bush K, Kivlahan DR, McDonell MB, Fihn SD, Bradley KA (1998). The AUDIT alcohol consumption questions (AUDIT-C): an effective brief screening test for problem drinking. Ambulatory care quality improvement project (ACQUIP). Alcohol use disorders identification test. Arch Intern Med.

[40] Bradley KA, DeBenedetti AF, Volk RJ, Williams EC, Frank D, Kivlahan DR (2007). AUDIT-C as a brief screen for alcohol misuse in primary care. Alcohol Clin Exp Res.

[41] Bradley KA, Bush KR, Epler AJ, Dobie DJ, Davis TM, Sporleder JL, Maynard C, Burman ML, Kivlahan DR (2003). Two brief alcohol-screening tests from the alcohol use disorders identification test (AUDIT): validation in a female veterans affairs patient population. Arch Intern Med.

[42] Berman AH, Bergman H, Palmstierna T, Schlyter F (2005). Evaluation of the drug use disorders identification test (DUDIT) in criminal justice and detoxification settings and in a Swedish population sample. Eur Addict Res.

[43] Kessler RC, Barker PR, Colpe LJ, Epstein JF, Gfroerer JC, Hiripi E, Howes MJ, Normand SL, Manderscheid RW, Walters EE, Zaslavsky AM (2003). Screening for serious mental illness in the general population. Arch Gen Psychiatry.

[44] Hu SS, Neff L, Agaku IT, Cox S, Day HR, Holder-Hayes E, King BA (2016). Tobacco product use among adults - United States, 2013-2014. MMWR Morb Mortal Wkly Rep.

[45] Mendelsohn CP, Gartner C (2015). Electronic cigarettes. What should you tell your patients?. Med Today.

[46] Moyses C, Hearn A, Redfern A (2015). Evaluation of a novel nicotine inhaler device: part 2—effect on craving and smoking urges. Nicotine Tob Res.

[47] Stead LF, Hartmann-Boyce J, Perera R, Lancaster T. Telephone counselling for smoking cessation. Cochrane Database Syst Rev. 2013;8:Cd002850. 10.1002/14651858.CD002850.pub3 23934971

[48] Clare P, Slade T, Courtney RJ, Martire KA, Mattick RP (2014). Use of smoking cessation and quit support services by socioeconomic status over 10 years of the National Drug Strategy Household Survey. Nicotine Tob Res.

[49] Miller C, Wakefield M, Roberts L (2003). Uptake and effectiveness of the Australian telephone Quitline service in the context of a mass media campaign. Tob Control.

[50] Wilson A, Guillaumier A, George J, Denham A, Bonevski B (2017). A systematic narrative review of the effectiveness of behavioural smoking cessation interventions in selected disadvantaged groups (2010-2017). Expert Rev Respir Med.

[51] SRNT Subcommittee on Biochemical Verification. Biochemical verification of tobacco use and cessation. Nicotine Tob Res. 2002;4:149–59. 10.1080/14622200210123581 12028847

[52] Cheung KL, de Ruijter D, Hiligsmann M, Elfeddali I, Hoving C, Evers S, de Vries H (2017). Exploring consensus on how to measure smoking cessation. A Delphi study. BMC Public Health.

